# Contemplative Practice and Responses to Mortality Salience: Evidence from Long-Term Sufi Meditators

**DOI:** 10.3390/bs16081420

**Published:** 2026-08-18

**Authors:** Elisa Puvia, Luca Simione, Nima Gholizadeh, Shovaneh Feyzi, Antonino Raffone

**Affiliations:** 1 Department of Psychological and Social Sciences, John Cabot University, 00165 Rome, Italy; 2 Department of Psychology, Università degli Studi Internazionali di Roma-UNINT, 00147 Rome, Italy; 3 Department of Psychology, Sapienza University of Rome, 00185 Rome, Italy

**Keywords:** mortality salience, Terror Management Theory, Sufism, contemplative practice, existential defensiveness, death cognition

## Abstract

Mortality salience research has played a central role in understanding responses to death awareness and existential threat. At the same time, relatively little research has examined whether long-term contemplative practice may shape affective, cognitive, and implicit responses to mortality-related stimuli. The present study investigated these processes in long-term contemplative practitioners from a Sufi tradition in which reflection on death occupies a central role. Participants completed an implicit measure of self- versus other-oriented attitudes before and after a mortality salience induction, along with self-report measures of anxiety, affect, and death-thought accessibility. Following mortality reminders, non-meditators reported higher anxiety and showed a relative increase in implicit prosocial orientation, consistent with defensive coping responses described in Terror Management Theory research. In contrast, long-term contemplative practitioners exhibited lower anxiety, a greater accessibility of death-related thoughts, and no increase in prosocial orientation following mortality salience. Greater meditation experience was associated with lower anxiety and negative affect. These findings suggest that sustained contemplative engagement may be associated with reduced existential defensiveness and more open engagement with death-related cognition. More broadly, the study highlights the value of investigating long-term contemplative practitioners in mortality salience research and points toward new directions for research on meditation, existential cognition, and adaptive responses to mortality awareness.

## 1. Introduction

While both the time and how an individual dies are usually unpredictable (apart from cases of suicide), the fact that death occurs is inescapable. Terror Management Theory (TMT; for a review, see Pyszczynski et al., 2015; Greenberg et al., 1997) posits that the awareness of the inevitability of death is a critical motivating force in human cognition and behavior. The theory maintains that people cope with their fear of death via two cultural anxiety-buffering mechanisms, including shared beliefs about reality that imbue the world with meaning and structure (cultural worldviews), and the sense of worth derived from meeting the standards of those worldviews (self-esteem).

Research derived from TMT has extensively documented that mortality reminders often elicit defensive responses that protect cultural worldviews, promote ingroup-favoritism, and justify prejudice or hostility toward threatening outgroups (e.g., Arrowood & Cox, 2020). At the same time, TMT research has recognized that these responses are not uniform, but depend on individual and contextual factors, including the availability of existential resources such as self-esteem, cultural orientation, and other psychological characteristics (e.g., Arndt et al., 2002; Burke et al., 2010; Park & Pyszczynski, 2016; Solomon et al., 2026). An emerging line of research has begun to examine whether awareness of mortality may also foster constructive psychological outcomes under some conditions. Rather than exclusively producing defensive reactions, mortality awareness has been associated with greater meaning seeking, prosociality, personal growth, and other more adaptive responses (e.g., Vail et al., 2012).

Building on this emerging literature, researchers have also turned their attention to the role of contemplative practices in shaping the responses to mortality awareness. For example, mindfulness-based approaches have been associated with a reduced avoidance of threatening thoughts and less defensive responses following mortality reminders (e.g., Park & Pyszczynski, 2019). However, this line of research has primarily focused on relatively brief interventions or forms of contemplative practice that do not explicitly engage with mortality. Much less attention has been devoted to contemplative traditions in which sustained reflection on death constitutes an integral component of practice (but see Alparslan & Ersin Kuşdil, 2025). Such traditions may therefore provide a particularly informative context for examining whether long-term, intentional, and culturally structured engagement with mortality is associated with less defensive responses to mortality awareness.

To address this gap, the present study focuses on long-term Sufi meditators, a contemplative tradition in which awareness of death occupies a central place in spiritual practice. Specifically, we examine whether sustained contemplative engagement with mortality is associated with distinctive affective, cognitive, and implicit responses to existential threat. By doing so, the present research seeks to contribute to the growing literature on the adaptive potential of mortality awareness while also extending the TMT framework by examining one possible source of enduring existential resources associated with less defensive responses to mortality.

### 1.1. Contemplative Practices and TMT

A growing body of research (e.g., Schultz & Arnau, 2019; Piotrowski et al., 2020; Routledge & Juhl, 2010) has shown that contemplative practice experience is associated with reduced defensiveness responses after mortality reminders and predicts prosocial behavior in intergroup contexts (Berry et al., 2023).

Preliminary evidence suggests that mindfulness, whether conceptualized as a dispositional trait or temporarily induced through meditation, may attenuate defensive responses to mortality salience. Niemiec et al. (2010) found that dispositional mindfulness—a trait characterized by receptive attention to present-moment experience—was associated with lower levels of defensive reactions to reminders of death, such as a reduced acceptance of prejudice against an outgroup (Study 2) and increased self-enhancement strivings (Study 4). Individuals high in mindfulness also showed an increase in death-related thoughts following mortality salience, an effect not observed among those lower in mindfulness. Similarly, Park and Pyszczynski (2019, Studies 2 and 3) reported that a brief, one-time meditation training administered prior to the experimental session eliminated the effects of mortality salience on worldview defense and reduced the suppression of death-related thoughts. Taken together, these findings suggest that mindfulness may reduce the tendency to avoid threatening cognitions, potentially because it promotes a more accepting and nonreactive awareness of one’s thoughts. However, most of the existing evidence derives from mindfulness-based approaches that do not explicitly center on mortality itself, leaving open the question of whether similar patterns may emerge in contemplative traditions where death is an explicitly cultivated theme. Furthermore, while these findings document the positive effects of mindfulness both as a trait and following short-term experimental induction, the long-term effects of meditation on existential defensiveness remain underexplored.

Building on evidence that mindfulness fosters a more open engagement with mortality, we sought to extend this inquiry to a contemplative tradition that explicitly and deliberately focuses on mortality. Among belief systems that emphasize a peaceful relationship with death, Sufism—a contemplative and mystical branch of Islam—stands out for its central focus on the spiritual meaning of dying. Sufi thought encourages individuals to approach death not as a threat to be avoided, but as a reunion with the divine. Death is described as the “wedding night”, a metaphor for union with God (Alparslan & Ersin Kuşdil, 2025). From this perspective, death is not feared but longed for and is regularly commemorated as a moment of transcendence. This theological and emotional orientation toward mortality may provide practitioners with a powerful existential resource, enabling them to manage thoughts of death without the need for defensive psychological responses.

A further unresolved issue concerns the long-term effects of sustained engagement in contemplative practices that explicitly embrace mortality. Therefore, the present study investigates whether regular participation in a contemplative practice that explicitly embraces death is associated with reduced existential defensiveness. Given the established link between meditation and mental health (Pan et al., 2024), we also controlled for participants’ overall psychological well-being.

### 1.2. Meaning in Life and Spiritual Transcendence as a Buffer Against Existential Concerns

In recent years, additional psychological buffers to existential concerns have been proposed. One factor is the presence of meaning in life, or the extent to which individuals perceive their lives as coherent, purposeful, and significant. Individuals lacking this existential resource increased their levels of death anxiety after mortality salience (MS), whereas those high in meaning appear more resilient (Routledge & Juhl, 2010). Having a meaningful life also implies being part of something larger, more enduring, and of greater significance than one’s physical existence providing feelings of death transcendence. Thus, another related buffer against MS is spiritual transcendence. Research has proved that spiritual transcendence reduces the effect of mortality salience on increased altruistic tendencies among religious (i.e., Catholics) and nonreligious (i.e., atheists) people (Piotrowski et al., 2020), as well as among non-WEIRD, largely unreligious Easterners (Dong et al., 2018). All in all, the findings reviewed above suggest that people are less prone to showing defensive responses to mortality salience as a result of the beneficial impact of dispositional variables, like the presence of meaning in life, and spiritual transcendence. Importantly, this effect does not depend on the belief systems from which it arises.

### 1.3. TMT and Prosocial Behavior

A growing body of evidence indicates that mortality concerns can foster prosocial inclinations. Jonas et al. (2002) coined the term “Scrooge effect” to describe a constructive consequence of mortality salience: much like Dickens’s protagonist, who responded to the prospect of an insignificant and lonely death by becoming kinder and more generous, individuals may seek to manage death anxiety by engaging in benevolence. From a TMT perspective, this shift can be understood as an attempt to uphold cultural values that enhance self-esteem and provide existential protection.

Scholars have identified several factors that clarify when mortality salience promotes or inhibits prosocial behavior. One concerns the specific content of cultural worldviews. Because cultural prescriptions often contain contradictory norms—such as those promoting generosity alongside those encouraging self-interest—MS effects can vary, helping to explain the inconsistent findings regarding whether MS fosters prosociality or greed (Jonas et al., 2013; Gailliot et al., 2008).

Beyond normative ambiguity, the nature of the prosocial cause itself further shapes responses to mortality salience. Helping behaviors that indirectly affirm symbolic meaning—such as charitable donation—often increase under MS, whereas behaviors that highlight the body’s vulnerability—such as organ donation—tend to decrease, as they undermine symbolic defenses against death (Hirschberger et al., 2008).

Beyond cultural prescriptions and situational features, individual differences also shape prosocial responses to MS. Joireman and Duell (2005) showed that individuals oriented toward maximizing their own gains shifted toward self-transcendence values under MS, whereas dispositionally prosocial individuals were less affected. Extending this line of work, Dong et al. (2018) found that individuals low in spirituality were particularly likely to shift toward prosocial attitudes following mortality reminders.

Together, these findings suggest that mortality salience can foster prosociality, but its expression depends on a complex interplay between cultural prescriptions, situational cues, and personality traits. From this perspective, prosocial orientation may function either as a defensive response to existential threat or as an expression of reduced defensiveness, depending on individuals’ existential resources. This distinction motivated the use of an implicit measure of self- versus other-oriented attitudes in the present study.

### 1.4. The Present Study

Building on the body of work discussed above, the present study aims to make a novel contribution by examining the role of long-term contemplative practices in managing existential concerns via a quasi-experimental design. Rather than testing the presence or absence of a uniform mortality salience effect, the present study adopts an individual-differences approach, examining whether long-term engagement in a contemplative tradition that explicitly integrates death is associated with distinct patterns of responding to mortality reminders. Specifically, it investigates whether experienced Sufi meditators, known for their deep consideration of death, exhibit defensive responses to mortality salience. Additionally, the study explores Sufis’ responses to mortality salience compared to those of lay people without prior meditation experience. Defensive responses are operationalized as an implicit bias toward other- versus self-interest (i.e., prosocial orientation), assessed before and after MS induction.

Within the domain of prosocial attitudes, short-term mindfulness training has been shown to increase helping behaviors toward outgroup members (Berry et al., 2023). Furthermore, contemplating one’s own mortality has been associated with increased prosocial attitudes (Moon, 2019) and values (Joireman & Duell, 2005). However, little is known about the effects of long-term contemplative practice on defensive reactions to MS, particularly in relation to prosocial orientations.

Sufism offers a distinctive context for addressing this gap. With its special emphasis on death, conceived as a joyful experience to be embraced and contemplated, Sufism provides a unique case for investigating the potential benefits of a sustained confrontation with mortality. Moreover, Sufism integrates both the personal and group-based dimensions of spiritual development. This dual emphasis makes Sufism a particularly relevant case for the present investigation, given that our implicit measure directly targets the balance between other- and self-oriented interests—a dynamic that is central to its spiritual practice.

Beyond our primary interest in examining how contemplative practice shapes coping with thoughts of death, we also drew on the extensive literature on the cognitive processes underlying defensive responses to mortality salience (Arndt et al., 2004). This framework allowed us to explore the potential mechanisms through which meditation may exert its effects. In particular, we investigated whether a lower tendency to suppress unpleasant thoughts—a process often fostered by meditation—would be reflected in higher death-thought accessibility (DTA), and how this, in turn, would relate to affective states and defensive responses following mortality salience.

Our specific hypotheses are as follows:

**H1.**  *Following mortality salience, long-term Sufi meditators are expected to report lower levels of state anxiety and negative affect compared to non-meditators.*

**H2a.**  *The effects of mortality salience on participants’ affective responses are expected to correlate with dispositional variables such as the presence of meaning in life, satisfaction with life, mental health, and spiritual transcendence.*

**H2b.**  *Among Sufi meditators, the strength of such effects would also correlate with reported years of practice.*

**H3.**  *Among non-meditators, reminders of mortality are expected to be associated with an increased implicit positive evaluation of other-related compared to self-related information from pre- to post-MS induction.*

**H4a.**  *Due to their sustained engagement with death contemplation and their dual focus on both personal and group-based dimensions, Sufi meditators are expected to be less affected by MS. They will not show increased prosocial orientation following mortality salience but will endorse self- and other-related information equally.*

**H4b.**  *Alternatively, experienced meditators may show a preference for self-related information, consistent with evidence of a self-relevance processing advantage (for a review, see Sui & Humphreys, 2017) even among long-term meditators (Trautwein et al., 2016)—moreover, because of the nature of Sufism with its special focus on the self and death as a way towards a reunion with God.*

**H5.**  *Following mortality salience, non-meditators are expected to show lower levels of death-thought accessibility, whereas Sufi meditators are expected to show higher levels of DTA.*

## 2. Materials and Methods

### 2.1. Participants

An a priori power analysis was conducted using G*Power (version 3.1.9.7; Heinrich-Heine-Universität, Düsseldorf, Germany) for a repeated-measures ANOVA with a within–between interaction. Parameters were set as follows: effect size (f) = 0.20 (small to medium), significance level (α) = 0.05, statistical power (1 − β) = 0.80, two groups, and two measurement occasions. The analysis indicated that a total sample size of about 52 participants is required to achieve the specified power, corresponding to approximately 26 participants per group. To account for data loss and attrition, we enrolled sixty-seven participants from the provinces of Mazandaran and Gilan, northern Iran. All volunteered to participate in this study. They did not receive credits or money for their participation. Since the aim of our study was to compare the joint effect of long-term meditation along with personality traits in managing existential concerns, a sample of 31 Iranian Sufism practitioners (10 female and 21 males) and a sample of 36 Iranians with no meditation experience (23 female and 13 males) were recruited. Twelve participants were excluded from further analyses: nine participants failed to complete all the experimental sessions or the questionnaires, and three participants were removed due to technical problems during data collection that caused data loss. The final sample consisted of 55 participants (82% of the original sample).

Twenty-four were Iranian Sufism practitioners (seven female and eighteen male); their age ranged from 19 to 84 years old (*M* = 43.17; *SD* = 16.27). Inclusion criterion for this group was a lifetime meditation experience of more than 1000 h (or approximately three years). Their lifetime meditation experience ranged from 1 to 39 years (*M* = 15.62; *SD* = 9.40).^1^

There were 31 participants (20 female and 11 male) with no meditation experience aged between 22 to 56 (*M* = 36.97; *SD* = 8.40). Inclusion criterion for this group was reporting no or only sporadic meditation experience (less than 100 h lifetime). All indicated not having any a priori meditation experience.

### 2.2. Procedure and Material

Participants were recruited through personal contacts of two research assistants, NG and SF, and by snowball sampling. Participants were informed that the study was about their perspectives on life, lifestyle, and personality traits. Informed consent was obtained prior to the experiment, and all participants were debriefed following the completion of the study. In the first part of the study participants were presented with a package of trait questionnaires, assessing their level of spirituality, overall level of mental health, and their perceived presence of meaning in life. In this session, also demographic variables and meditative expertise were collected. Participants completed the trait questionnaires independently, in paper-and-pencil format, following the prescribed order. A research assistant was available to answer questions or clarify instructions if needed, but participants completed the questionnaires at their own pace, and no time limit was imposed. The experimental procedure was conducted and supervised by a research assistant. It started with the baseline assessment of the Affective Misattribution Procedure (AMP) measure followed by the MS manipulation. Afterwards, participants completed two filler questionnaires, State-Trait Anxiety Inventory Form (STAI-Y; Abdoli et al., 2020; Spielberger et al., 1983) and the Positive and Negative Affect Scale (PANAS; Watson et al., 1988), to measure participants’ levels of anxiety following death reminders and positive and negative affect, respectively. These distractors were included because previous studies have shown that mortality salience effects occur after people have been distracted from thoughts of their own death (Greenberg et al., 1994); and to rule out the possibility that mood or anxiety and not terror management processes were responsible for the findings of this research. This delay is followed by the second assessment of the AMP, followed by a word completion task to assess levels of death-thought accessibility (Greenberg et al., 1994). The present study was not preregistered. Validated Persian versions were used for all standardized questionnaires, with the exception of the Self-Transcendence subscale, for which no validated Persian version was available. This subscale was translated specifically for the present study by the two Persian-speaking research assistants and reviewed by the research team to preserve the meaning of the original items as closely as possible. All the measures were presented in the following order.

### 2.3. Trait Questionnaires

Self-transcendence. Participants’ level of self-transcendence was assessed through the Self-Transcendence subscale from the Temperament and Character Inventory questionnaire (Cloninger et al., 1994). Twenty-six items were employed to assess participants’ level of self-transcendence along three dimensions: self-forgetful vs. self-conscious experience (10 items), transpersonal identification vs. self-differentiation (8 items), and spiritual acceptance vs. rational materialism (8 items). Answers were given on a 5-point Likert scale from 1 (absolutely false) to 5 (absolutely true). Higher scores indicate higher levels of self-transcendence. Internal reliability was good in our sample (Cronbach’s α = 0.84, McDonald’s ω = 0.86).

Mental health. Participants’ level of mental health was assessed through two questionnaires. First, the psychological distress was assessed using the Persian version of the Depression Anxiety Stress Scale-21 Items (DASS-21; Habibi et al., 2017; Kakemam et al., 2022; Lovibond & Lovibond, 1995). This scale includes 21 items divided into three 7-item subscales. This self-report questionnaire measures the prevalence of depression (e.g., “I felt downhearted and blue”), anxiety (e.g., “I felt scared without any good reason”), and stress (e.g., “I found myself getting agitated”) signs and symptoms. Participants responded to these items on a 4-point Likert scale from 0 (it does not apply to me at all) to 3 (it applies to me most of the time). A total score representing general psychological distress was computed as the sum of all items; higher scores represent higher psychological distress. In the present study, the overall score showed a good internal consistency (Cronbach’s α = 0.83, McDonald’s ω = 0.84). Second, we used the Persian version of the Satisfaction With Life Scale (SWLS; Diener et al., 1985; Nooripour et al., 2021) as an indicator of psychological functioning and emotional well-being. This 5-item scale assesses individuals’ cognitive evaluations of their overall life satisfaction (e.g., “I am satisfied with my life”). Each item has been scored on a 7-point Likert scale from 1 to 7. In the present sample, the scale demonstrated good internal consistency (Cronbach’s α = 0.84, McDonald’s ω = 0.85).

Meaning in life. The Iranian version of the Meaning in Life Questionnaire (MLQ; Mesrabadi et al., 2013; Steger et al., 2006) was used to assess participants’ perceived presence of meaning in life (e.g., “My life has a clear sense of purpose”). Only the 5 items of the subscale Presence of meaning were retained. Each item was rated on a 7-point Likert scale from 1 (absolutely untrue) to 7 (absolutely true). In the present study, the internal consistency of the two scale was good for both the presence of meaning (Cronbach’s α = 0.77, McDonald’s ω = 0.80) and the search for meaning component (Cronbach’s α = 0.89, McDonald’s ω = 0.90).

### 2.4. First Experimental Phase

Affective Misattribution Procedure (AMP). The procedure of the AMP largely followed the general recommendations by Payne et al. (2005). Participants were seated in front of a computer and informed that the study examined “how people make simple but quick judgements”. Participants were told that they would see a word followed by a Chinese character, flashed one after the other (for a similar procedure, see Sava et al., 2012). They were told that the word label simply served as a warning signal for the Chinese character and that they should do nothing with the word attribute. Instead, their job was to judge the visual pleasantness of each Chinese pictograph. In addition, participants were instructed to respond quickly. Participants were told that the word attributes can sometimes bias people’s responses to the Chinese ideographs, and that they should try their absolute best not to let the words bias their judgments of the Chinese ideographs in any possible way.

On each trial of the task, participants were first presented with a fixation cross for 500 ms, which was replaced by a prime stimulus of either other- or self-interest words for 75 ms. The presentation of the prime was followed by a blank screen for 125 ms, after which a Chinese ideograph appeared for 100 ms. The Chinese ideograph was then replaced by a black-and-white pattern mask, and participants were asked to make their response. Participants’ task was to indicate whether they consider the Chinese ideograph as visually more pleasant or visually less pleasant than the average Chinese ideograph using a key on the computer keyboard. The particular key assignment for a given trial appeared on the screen together with the black-and-white patter mask that replaces the Chinese ideograph. The pattern mask remains on the screen until participants gave their response. The next trial started after an intertrial interval of 500 ms. Participants first completed 10 practice trials using neutral control words to familiarize themselves with the computerized procedure before beginning the experimental trials. As prime stimuli, five self-interest (keep, gain, earn, profit, and obtain) and five other-interest (i.e., share, give, aid, favor, and assistance) word attributes were used and translated into Persian for use in the present study. These attributes were taken from the SOI-IAT measure (Thornton & Aknin, 2020), which evaluates participants’ implicit bias toward others- vs. self-interest (i.e., prosociality). Each prime was presented 10 times, summing up to a total of 100 trials (for a similar procedure, see Gawronski & Ye, 2014). As target stimuli, we use 10 Chinese ideographs. Order of trials and prime- target combinations were randomized by the computer for each participant. During data collection, no substantial difficulties related to computer use were observed by the research assistant. Data were treated following the original instructions by Payne et al. (2005).

MS manipulation. Participants responded to two open-ended questions (used in prior TMT studies; e.g., Cox et al., 2019): “Please briefly describe the emotions that the thought of your own death arouses in you,” and “Jot down, as specifically as you can, what you think will happen to you as you physically die and once you are physically dead.” Participants were instructed to answer with their first, natural response to each of these questions. Previous research (e.g., Greenberg et al., 1994) has shown that the effects of an explicit MS prime are more robust after a short delay when death-related thought is accessible, but out of focal attention. For this reason, two filler questionnaires were included between MS manipulation and the administration of the dependent variables.

### 2.5. State Questionnaires

Delay/Affect. Two state questionnaires were used measuring distress and emotional state. Specifically, the Persian version of the State-Trait Anxiety Inventory Form (STAI-Y; Abdoli et al., 2020; Spielberger et al., 1983), and of the Positive-And-Negative Affective Scale (PANAS; Watson et al., 1988). STAI-Y measures the anxiety and psychological distress (e.g., “I am tense”, and “I feel frightened”) as the sum of 20 items (higher scores indicated higher anxiety state), rated on a 5-point Likert scale from 1 to 5. PANAS consisted of 10 positive (e.g., “Enthusiastic”) affect items (PA; Cronbach’s α = 0.83, McDonald’s ω = 0.84) and 10 negative (e.g., “Distressed”) affect items (NA; Cronbach’s α = 0.85, McDonald’s ω = 0.86).

### 2.6. Second Experimental Phase

AMP. A second assessment of the AMP was administered. This version was identical to the one used in the first phase of the experiment.

Death-Thought Accessibility (DTA). To assess death-thought accessibility, participants completed a set of 20-word fragments containing a blank space into which letters could be written (Webber et al., 2016). Of the 20-word fragments, six could be completed with either a death-related word or a neutral word. For example, the word-fragment, “SK_ _L” could be completed as the neutral word skill or as the death-related word skull. Possible death-related words include buried, dead, grave, killed, skull, and coffin. The Persian version of the DTA task was developed by adapting the original English word fragments into Persian while preserving the ambiguity of each fragment. For example, the Persian word for “death” (marg) was presented as “_arg”, which could also be completed as barg (“leaf”). Participants were informed that this task examines their first, natural response, and, thus, they were instructed to complete each fragment quickly, with the first word that comes to mind. Death-thought accessibility score was calculated by summing the number of death-related completions, potentially ranging from 0 to 6.

## 3. Results

As an initial step, we examined whether the two groups differed on the dispositional variables assessed during the first session, to ensure comparability at baseline. To this end, we conducted a series of Welch’s *t*-tests, with the results reported in Table 1. The analysis revealed significant group differences in self-transcendence and the presence of meaning in life. In contrast, no significant group differences emerged for any of the DASS subscales (all *p*s > 0.18), nor for the overall DASS score or life satisfaction. These findings suggest that the two groups did not differ in baseline levels of psychological distress before the mortality salience manipulation and that the subsequent findings cannot be attributed to pre-existing differences in depression, anxiety, or stress. We also compared the two groups on gender distribution and found a significant difference in the number of women and men in the sample, χ^2^(1) = 6.76, *p* < 0.05. Consistently, gender was included as a covariate in subsequent analyses.

### 3.1. MS Induction Effects on Affective State

**Figure 1:**
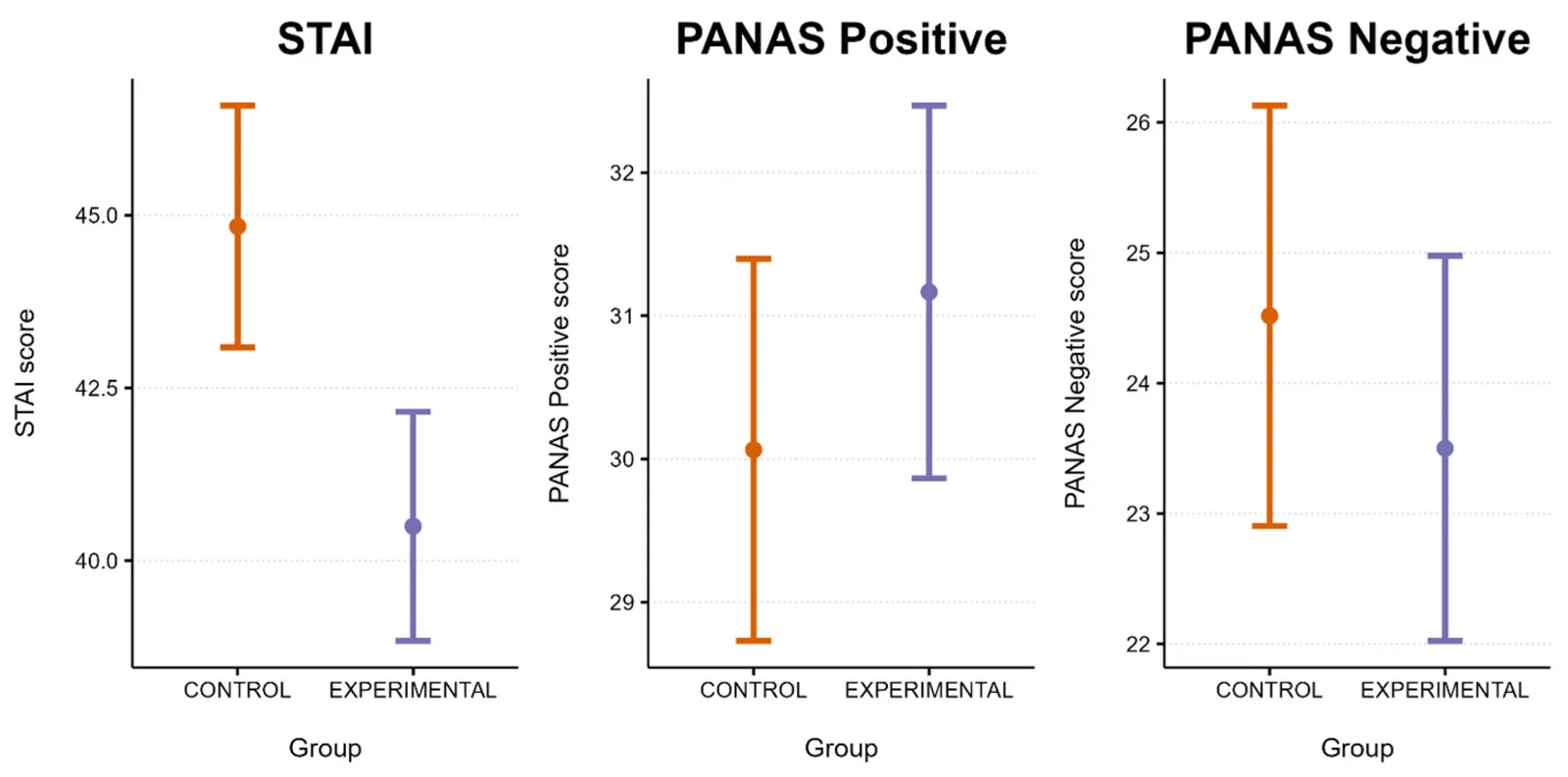
Mean scores of STAI, PANAS Positive, and PANAS Negative for the two groups.

To test H1, we examined whether Sufi meditators differed from non-meditators in their affective state following the mortality salience induction. Figure 1 displays the mean scores for STAI, PANAS Positive, and PANAS Negative across groups. We conducted a one-way MANCOVA with the groups (Sufi meditators vs. non-meditators) as the independent variable and STAI, PANAS Positive, and PANAS Negative as dependent variables, controlling for age, gender, mental health, satisfaction with life, self-transcendence, and meaning in life. The analysis revealed a trend-level effect of group, Wilks’ lambda = 0.82, *F*(4, 44) = 2.35, *p* = 0.07. Follow-up univariate analyses showed a significant group difference on state anxiety, *p* < 0.05, such that the non-meditators reported higher levels of state anxiety after the MS induction than Sufi meditators. No significant effects emerged for PANAS Positive, *p* = 0.57, and PANAS Negative, *p* = 0.50.

To test hypothesis H2a, the same MANCOVA also revealed an overall significant effect of the dispositional factors of MLQ, Wilks’ lambda = 0.68, *F*(4, 44) = 5.14, *p* < 0.01, SWLS, Wilks’ lambda = 0.52, *F*(4, 44) = 10.17, *p* < 0.001, and DASS Tot, Wilks’ lambda = 0.51, *F*(4, 44) = 10.41, *p* < 0.001, whereas no significant effect of self-transcendence emerged, *p* = 0.80. To further probe this pattern of results, we correlated the psychological dispositional factors with the affective states after mortality salience induction. The analysis revealed that MLQ and SWLS were positively correlated with PANAS Positive, respectively, *r* = 0.35 and *r* = 0.34, *p*s < 0.01, and negatively correlated with STAI, respectively, *r* = 0.37 and *r* = 0.57, *p*s < 0.01. SWLS was also negatively correlated with PANAS Negative, *r* = −0.32, *p*s < 0.05. Participants’ level of psychological distress as assessed with the DASS significantly correlated with STAI, *r* = 0.74, *p* < 0.01, PANAS Negative, *r* = 0.57, *p* < 0.01, and PANAS Positive, *r* = −0.35, *p* < 0.05. Of note, self-transcendence did not correlate with any of the outcomes considered.

**Figure 2:**
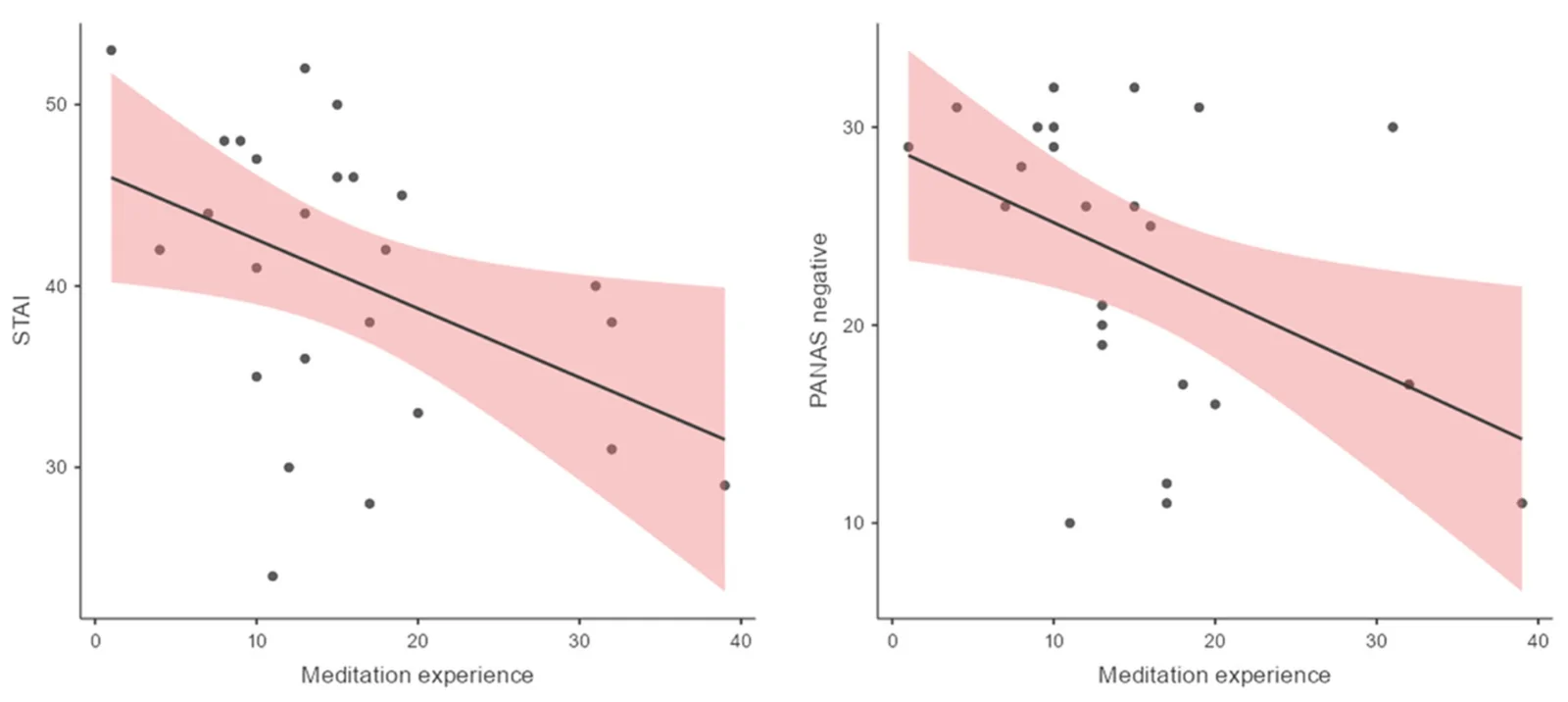
Scatterplots of the correlation between meditation experience (years) and STAI (**left**) and PANAS Negative (**right**). The shaded area represents the standard error.

Lastly, we conducted a set of correlational analysis on the relationship between STAI and PANAS scores and meditative experience (in years) within the Sufi meditators group (H2b). Meditation experience was negatively correlated with both STAI, *r* = −0.44, *p* < 0.05, and PANAS negative, *r* = −0.47, *p* < 0.05 (see Figure 2), but not with PANAS positive, *r* = 0.24, indicating that more years of practice were associated with lower anxiety and negative affect in response to MS.

### 3.2. Analyses for Prosocial Orientation Attitude

**Figure 3:**
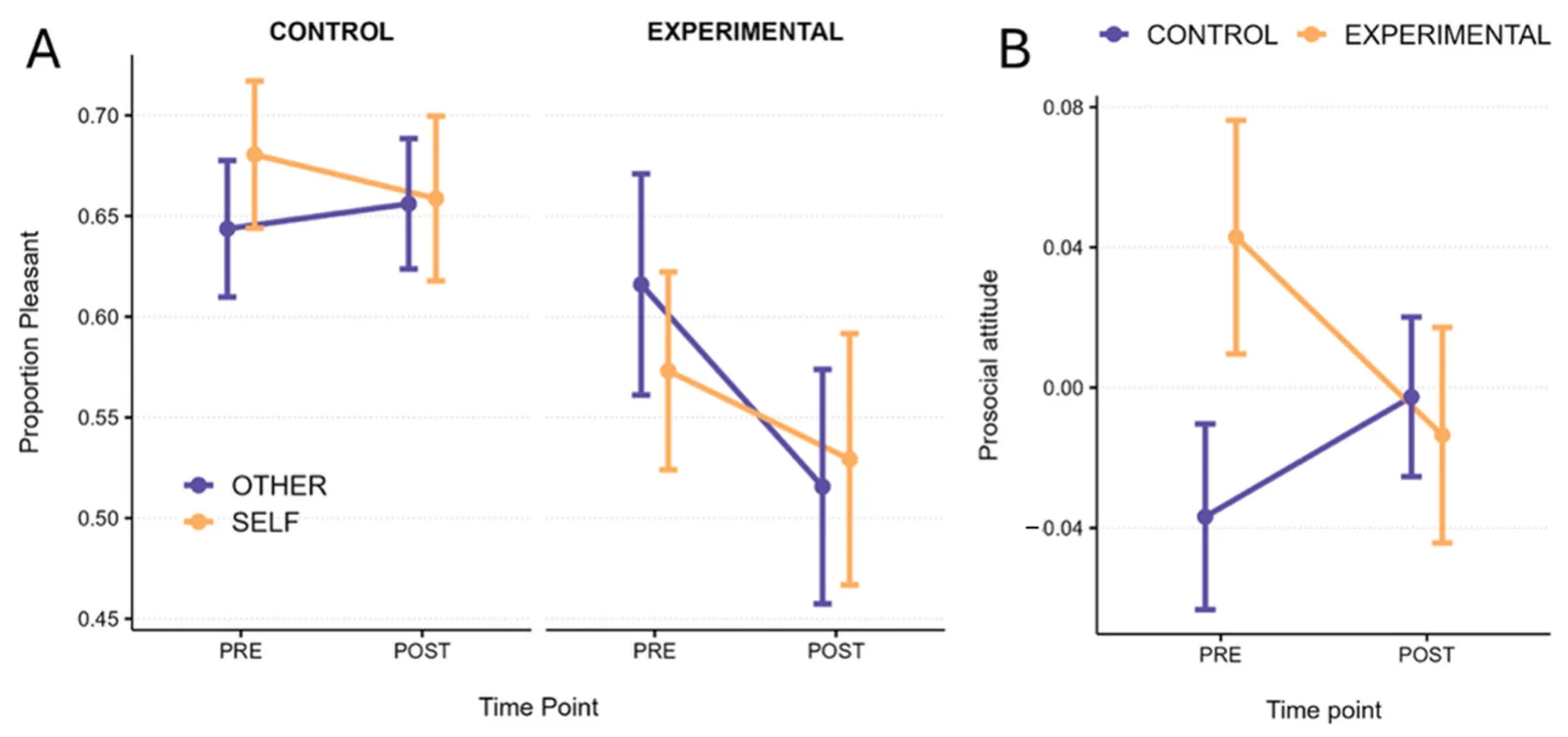
Panel (**A**): Proportion of pleasant responses in the AMP task for Sufi meditators and non-meditators before (PRE) and after (POST) mortality salience induction, separately for self-related (yellow) and other-related (blue) primes. Error bars represent standard errors. Panel (**B**): Prosocial orientation score in the AMP task for Sufi meditators and non-meditators before (PRE) and after (POST) mortality salience induction. Positive values indicate a higher proportion of pleasant responses to other-related primes. Error bars represent standard errors.

We then analyzed the results of the AMP before and after MS induction. Following the procedure used by Payne et al. (2005), trials with reaction times that were too fast (<100 ms reflecting anticipatory responding) or too slow (>2000 ms, reflecting inattentive responding) were removed from the analysis. For each participant, a pleasantness index was computed, defined as the proportion of pleasant responses for each prime type (self vs. other) and time of presentation (pre- vs. post-mortality salience induction). The proportion of pleasant responses across groups and conditions is reported in Figure 3A.

We then conducted a 2 (group: non-meditators vs. Sufi meditators) × 2 (prime: self vs. other) × 2 (time: pre and post induction) repeated-measures mixed ANOVA on this index. The analysis revealed a significant main effect of group, *F*(1, 208) = 8.74, *p* < 0.01, and marginally significant interaction between time and group, *F*(1, 212) = 3.09, *p* = 0.08. Given the exploratory nature of this study, we decomposed these significant effects through post hoc analyses. The group effect was due to a higher pleasantness proportion on average in the non-meditators group (*M* = 0.68) than in the long-term Sufi meditators group (*M* = 0.57). The interaction was qualified by significant contrasts, showing a lower proportion of pleasant responses for the meditators group at post-induction (*M* = 0.52) compared to the non-meditators group both at the pre- (*M* = 0.66), *t*(212) = 3.07, *p* < 0.01, and post-induction (*M* = 0.66), *t*(212) = 2.97, *p* < 0.01. All other contrasts were not significant, *p*s > 0.19.

We then computed a prosocial orientation bias for each participant, defined as the difference between the proportion of pleasant responses for other-interest prime words (share, give, aid, favor, and assistance) and that for self-interest prime words (keep, gain, earn, profit, and obtain). Higher and positive scores indicate a stronger proportion of pleasant responses for other-interest prime words, or a stronger prosocial orientation attitude. We computed this index before and after MS induction. The average prosocial orientation bias is reported in Figure 3B. It appears that the non-meditators increased this bias after MS induction, whereas the long-term Sufi meditators group decreased it. We then conducted another 2 (group: non-meditators vs. Sufi meditators) × 2 (time: pre- and post-induction) repeated-measures mixed ANOVA on the prosocial orientation bias. This analysis revealed a marginally significant main effect of time, *F*(1, 104) = 3.47, *p* = 0.06, and a significant interaction between time and group, *F*(1, 104) = 5.37, *p* < 0.05. Regarding the main effect of time, we found an overall lower prosocial orientation bias before (*M* = 0.002) than after MS induction (*M* = 0.007). Regarding the interaction, post hoc contrasts revealed a significant difference in prosocial orientation bias in the pre-induction AMP task between the two groups (Sufi meditators *M* = 0.042; and non-meditators: *M* = −0.037), *t*(106) = 2.01, *p* < 0.05. At post-induction, however, this difference was not significant (Sufi meditators: *M* = −0.014; non-meditators: *M* = −0.002), *p* = 0.78. Consistent with H3, non-meditators increased their positive evaluations of other-related (compared to self-related) information after mortality salience, suggesting an increase in prosocial orientation. In line with the H4b, after MS Sufi meditators increased instead their positive evaluations of self-related information and decreased their prosocial orientation, showing a preference for self-related information (Figure 3B).

To further explore the effect of the dispositional factors on prosocial orientation bias, we conducted a multiple regression analysis with the other-related bias as the outcome, the dispositional variables of DASS, SWLS, MLQ, and self-transcendence as the predictors, and the time and group as the independent variables, while controlling for age and gender. The analysis revealed that none of the considering factors reach significance in a model including both the control and experimental groups, *p*s > 0.24, nor in a model including the Sufi meditators only, *p*s > 0.09.

### 3.3. MS Induction Effects on DTA

**Figure 4:**
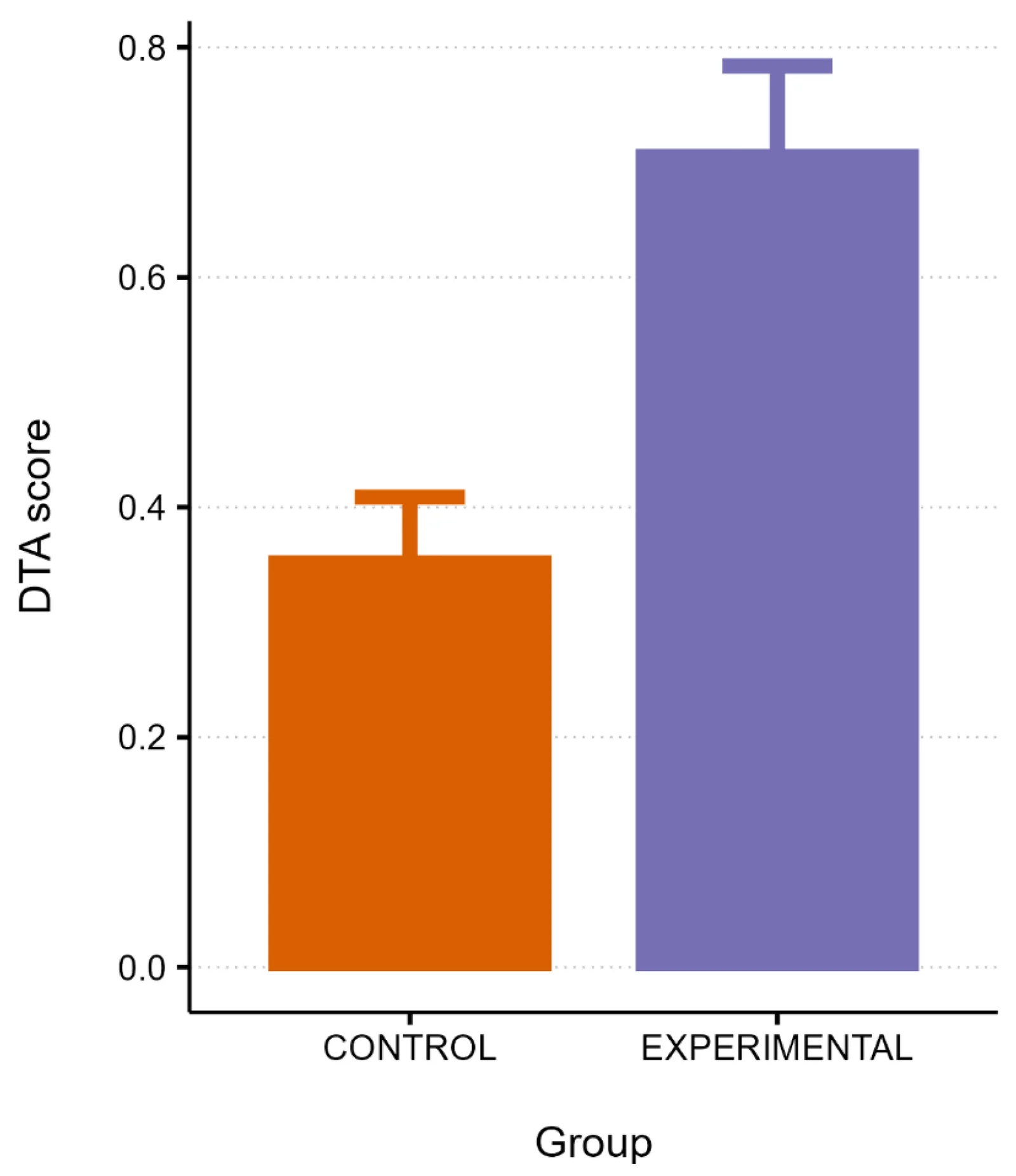
Mean number of death-related words reported in the DTA task by long-term Sufi meditators and non-meditators. Error bars indicate standard errors.

Death-thought accessibility (DTA) was used to evaluate the impact of mortality salience (MS) on death-related cognition in the two groups. Specifically, we conducted a one-way ANCOVA on the DTA scores (i.e., the number of death-related words reported), with group as the between-subject factor and age, gender, and affective state (i.e., STAI, PANAS Positive, PANAS Negative) as the covariates. Consistent with our H5, a significant main effect of the group emerged, *F*(1, 50) = 4.84, *p* < 0.05, according to which long-term Sufi meditators reported a higher number of death-related words (*M* = 0.75, *SE* = 0.14) compared to non-meditators (*M* = 0.33, *SE* = 0.12; see Figure 4). This result supports the idea that experienced meditators appear more attuned to death-related content without showing defensive avoidance.

## 4. Discussion

The present study investigated how individual characteristics, particularly long-term contemplative practice, are associated with different responses to mortality awareness. Although much of the empirical literature has examined defensive reactions to mortality awareness because of their importance for understanding prejudice, intergroup conflict, and worldview defense, Terror Management Theory has long recognized that mortality awareness can also promote adaptive responses depending on the existential resources available to the individual (e.g., Pyszczynski et al., 2015). The present study builds on and extends this line of research by examining whether engagement in a long-term contemplative tradition centered on a sustained reflection on mortality is associated with more adaptive responses to mortality awareness. Specifically, it addressed two important gaps in the literature. First, prior work has largely focused on short-term mindfulness inductions or dispositional mindfulness, leaving the long-term effects of sustained contemplative practice underexplored. Second, most existing research has examined meditation traditions that do not explicitly engage with mortality itself. By focusing on long-term Sufi meditators—a contemplative tradition in which reflection on death is central and explicitly cultivated—the present study contributes to a more nuanced understanding of when mortality awareness elicits defensive versus adaptive responses. Consistent with our hypotheses, Sufi meditators reported lower levels of state anxiety following mortality salience compared to non-meditators, suggesting that sustained contemplative engagement with mortality may buffer against anxiety-related responses to existential threat. Notably, this effect was specific to anxiety and did not extend to a general positive or negative affect, aligning with the idea that contemplative practice may selectively modulate existentially relevant emotional processes rather than global mood states. In line with prior research (Routledge & Juhl, 2010), individual differences in meaning in life, life satisfaction, and mental health were also associated with more adaptive affective responses following mortality salience, underscoring the role of existential and psychological resources in shaping responses to death-related threat. In contrast, self-transcendence did not show the expected associations with affective outcomes, suggesting that this construct may operate differently from other meaning-related resources or exert its influence through non-affective pathways. Beyond affective responses, we examined how mortality salience influenced the implicit prosocial orientation and cognitive accessibility of death-related thoughts. Although group differences in implicit prosocial orientation did not reach statistical significance, the observed pattern is theoretically informative. Prior to mortality salience, meditators showed a stronger other-oriented implicit bias than non-meditators. Following mortality salience, non-meditators exhibited a descriptive increase in prosocial orientation, consistent with the notion that prosociality may function as a defensive strategy for managing existential threat. In contrast, meditators showed a decrease in other-oriented bias, suggesting that prosocial orientation may be less necessary as a compensatory defense when existential threat is already integrated and less anxiety-provoking. Importantly, these patterns align with TMT accounts emphasizing that prosocial behavior may reflect either defensive or non-defensive processes, depending on individuals’ underlying existential resources. Consistent with this interpretation, Sufi meditators showed higher levels of death-thought accessibility following mortality salience. This finding converges with prior evidence showing that mindfulness and meditation reduce the suppression of death-related cognitions (Niemiec et al., 2010; Park & Pyszczynski, 2019) and supports the idea that sustained contemplative practice fosters a more open engagement with existential concerns. Rather than avoiding or defensively suppressing mortality-related thoughts, long-term meditators may tolerate and integrate such cognitions without the need for a compensatory worldview defense. Taken together, these findings contribute to a better understanding of the individual characteristics associated with different responses to mortality awareness. Consistent with the Terror Management Theory framework, these findings illustrate how enduring existential resources may shape responses to mortality awareness and further clarify the conditions under which mortality awareness may foster more adaptive rather than defensive responses.

### Limitations and Future Directions

Several limitations should be acknowledged. First, although the overall pattern of the findings was consistent with the theoretical expectations, some effects did not reach statistical significance. Future research with larger samples will be important to establish the robustness of these effects. Second, while the use of an implicit measure of self- versus other-oriented attitudes represents a methodological innovation, its interpretation warrants caution. Participants may not have perceived this distinction as directly relevant to worldview defense, potentially attenuating the observable effects. Future studies could strengthen measurement validity by combining implicit tasks with behavioral or self-report measures of prosociality and by validating the existential relevance of prime stimuli within specific cultural contexts. An additional limitation concerns the relatively wide age range among the long-term meditators. Although age was statistically controlled in the primary analyses and the two groups did not significantly differ in age at baseline, future studies should recruit larger samples that allow the respective contributions of age, accumulated life experience, and years of meditation practice to be examined separately.

A further limitation concerns the absence of a non-death-related control condition. Although the within-participant pre–post design allows us to examine changes following the mortality salience induction, it does not permit definitive conclusions regarding the specificity of the observed effects, as these changes might also be elicited by other emotionally salient experimental primes. Moreover, because death-thought accessibility was assessed only after the mortality salience manipulation, we cannot rule out the possibility that long-term meditators differ from non-meditators in the baseline accessibility of death-related thoughts. Future studies should include both a neutral control condition and a baseline assessment of DTA to more precisely isolate the effects of mortality salience.

## 5. Conclusions

Despite these limitations, the present study offers novel theoretical and methodological contributions. By examining a contemplative tradition that explicitly integrates mortality into its practice, the findings provide preliminary evidence that sustained engagement with death-related themes may attenuate existential defensiveness and promote more adaptive affective and cognitive responses. Methodologically, the combination of implicit measures with a quasi-experimental comparison between long-term meditators and non-meditators advances the existing approaches to studying terror management processes.

More broadly, the present work contributes to a growing body of research examining the individual and contextual conditions that shape responses to mortality awareness. The present findings suggest that long-term contemplative practice may represent one such enduring existential resource, associated with greater psychological openness, reduced anxiety, and a different pattern of cognitive engagement with mortality following mortality salience. Identifying these boundary conditions contributes to a more comprehensive understanding of terror management processes and helps clarify how existential awareness may support resilience and prosocial functioning under certain conditions.

Beyond their theoretical implications for Terror Management Theory, the present findings may also have practical relevance. A growing body of literature suggests that contemplative practices can promote psychological well-being and resilience through multiple psychological and physiological pathways (e.g., Hanley et al., 2015; Crosswell et al., 2024). The present findings also raise the possibility that changes in how individuals relate to mortality may represent one additional mechanism worthy of further investigation. Future longitudinal and intervention studies are needed to directly test this hypothesis. If confirmed, these findings may also inform interventions aimed at helping individuals cope more adaptively with existential concerns (Gandy, 2017).

## Figures and Tables

**Table 1:** Results of the Welch’s *t*-tests comparing the two groups on the dispositional variables (N = 55).

	Mean Long-Term Sufi Meditators	Mean Non-Meditators	Mean Difference	SE Difference	Statistic	df	Effect Size
Age	43.96	36.97	−6.99	3.62	−1.93	34.20	−0.54
Self transcendence	93.88	84.00	−9.88	3.33	−2.96 **	43.00	−0.81
MLQ	30.64	25.00	−5.64	1.45	−3.89 **	54.00	−1.03
DASS TOT	37.20	40.52	3.32	2.32	1.43	53.50	0.38
SWLS	22.28	19.84	−2.44	1.89	−1.29	46.20	−0.35

Note. The effects are reported with long-term Sufi meditators group as reference. Effect size is computed as Cohen’s d. df = degree of freedom; SE = standard error; DASS = Depression Anxiety Stress Scale, short version; MLQ = Meaning in Life Questionnaire, presence of meaning subscale; SWLS = Satisfaction With Life Scale. ** *p* < 0.01.

## Data Availability

The original data presented in the study are openly available in the Open Science Framework at https://osf.io/3kjmg/overview?view_only=d9715a63a549494fb99551e328c8ce51 (accessed on 6 August 2026).

## Note

1 Due to the difficulty of recruiting a sample of long-term meditators meeting these criteria, we included one participant with only one year of meditation experience. Importantly, this inclusion did not affect the main findings. All other meditators had at least four years of experience, well above the minimum threshold specified.
